# Integrins in Cardiovascular Health and Disease: Molecular Mechanisms and Therapeutic Opportunities

**DOI:** 10.3390/biom15020233

**Published:** 2025-02-06

**Authors:** Karolina Ławkowska, Klaudia Bonowicz, Dominika Jerka, Yidong Bai, Maciej Gagat

**Affiliations:** 1 Department of Histology and Embryology, Collegium Medicum in Bydgoszcz, Nicolaus Copernicus University in Torun, 85-092 Bydgoszcz, Poland; 2 Collegium Medicum, Mazovian Academy in Płock, 09-402 Płock, Poland; 3 Department of Cell Systems and Anatomy, University of Texas Health San Antonio, San Antonio, TX 78229, USA; baiy@uthscsa.edu

**Keywords:** integrins, cardiovascular diseases, vascular endothelial cells, vascular smooth muscle cells, platelets, cardiac fibroblasts, cardiomyocytes, integrin antagonists and antibodies, nanotherapy

## Abstract

Cardiovascular diseases, including atherosclerosis, hypertension, and heart failure, remain the leading cause of global mortality, with endothelial dysfunction and vascular remodeling as critical contributors. Integrins, as transmembrane adhesion proteins, are central regulators of cell adhesion, migration, and signaling, playing a pivotal role in maintaining vascular homeostasis and mediating pathological processes such as inflammation, angiogenesis, and extracellular matrix remodeling. This article comprehensively examines the role of integrins in the pathogenesis of cardiovascular diseases, focusing on their dysfunction in endothelial cells and interactions with inflammatory mediators, such as TNF-α. Molecular mechanisms of integrin action are discussed, including their involvement in mechanotransduction, leukocyte adhesion, and signaling pathways that regulate vascular integrity. The review also highlights experimental findings, such as the use of specific integrin-targeting plasmids and immunofluorescence to elucidate integrin functions under inflammatory conditions. Additionally, potential therapeutic strategies are explored, including the development of integrin inhibitors, monoclonal antibodies, and their application in regenerative medicine. These approaches aim not only to mitigate pathological vascular remodeling but also to promote tissue repair and angiogenesis. By bridging insights from molecular studies with their translational potential, this work underscores the promise of integrin-based therapies in advancing the management and treatment of cardiovascular diseases.

## 1. Introduction

Cardiovascular diseases, including atherosclerosis, hypertension, and chronic heart failure, are a major and growing global health issue. According to recent data, cardiovascular diseases remain the leading cause of mortality worldwide, accounting for approximately 17.9 million deaths annually, which constitutes 32% of all global deaths [1]. The incidence of these conditions has significantly increased over the past few decades, posing considerable challenges for healthcare systems [2]. Addressing these diseases effectively requires an in-depth understanding of the complex pathogenic mechanisms that underlie their progression.

A crucial factor in the development of cardiovascular diseases is the disruption of endothelial function [3]. The vascular endothelium is integral to maintaining systemic homeostasis, as it regulates vascular tone, blood flow, and inflammatory responses. When endothelial function is compromised, it leads to impaired vasodilation and heightened inflammatory and thrombotic activities [4]. Endothelial dysfunction is recognized as one of the initial stages of atherosclerosis, occurring well before clinical symptoms manifest. The inflammatory activation of the endothelium is associated with several molecular alterations that impact cell adhesive, migratory, proliferative, and chemotactic behaviors [5].

Integrins, transmembrane adhesion glycoproteins, are vital for endothelial function and vascular integrity. Composed of alpha (α) and beta (β) subunits, these heterodimeric proteins mediate cell adhesion, migration, signal transduction, and cell-matrix interactions by binding ligands such as fibrinogen, laminins, and collagen [6]. Within the vascular system, integrins regulate homeostasis, tissue repair, and angiogenesis, which is essential for wound healing and pathological processes in cardiovascular diseases [7].

Integrin dysfunction is implicated in conditions like atherosclerosis, where it disrupts cell adhesion and promotes plaque formation; hypertension, where it influences vascular remodeling; and chronic heart failure, where it impairs vasodilation and fosters inflammation [8]. Given their broad biological roles, integrins are promising therapeutic targets. Monoclonal antibodies and other inhibitors show potential in treating vascular diseases and limiting tumor angiogenesis, while regenerative medicine explores integrin modulation to enhance tissue repair [9].

Current research on integrin inhibitors aims to develop effective treatments for a range of vascular diseases. For example, monoclonal antibodies targeting specific integrins have shown the potential to inhibit angiogenesis in tumors, thereby limiting tumor growth and metastasis. Moreover, modulating integrin activity can support tissue healing and stimulate angiogenesis in regenerative medicine and tissue engineering [10].

In conclusion, integrins are critical to vascular health and disease. Further research into their mechanisms could pave the way for novel therapies, improving outcomes for patients with cardiovascular conditions [11].

The purpose of this article is not only to present the biological importance of integrin receptors within the vascular system but also to highlight recent scientific advances in understanding their role in regulating vascular processes. Additionally, we will explore potential therapeutic applications involving the modulation of integrin receptor activity, aiming to improve treatment outcomes in vascular diseases as well as to promote tissue repair and angiogenesis in regenerative therapies and tissue engineering.

## 2. The Importance of Vascular Endothelium 

### 2.1. Vascular Endothelium—The First Line of Defense

The vascular endothelium is a monolayer of cells lining the interior of blood vessels [12]. It plays a multifaceted role in maintaining vascular health, including regulating blood flow, controlling permeability across vessel walls, and initiating inflammatory responses [13]. As one of the most dynamic cellular layers in the body, the endothelium is crucial for the proper functioning of the circulatory system and immune defense [14].

Under normal conditions, the endothelium acts as a selective barrier, managing the exchange of substances such as nutrients, hormones, and waste products between the blood and tissues. However, when the endothelium is damaged or dysfunctional, these properties are altered, leading to vascular abnormalities and disease progression [15]. The vascular endothelium is vital in maintaining vascular integrity and acts as the first line of defense against various threats through its barrier, regulatory, and signaling functions. Table 1. summarizes the primary functions of the vascular endothelium, emphasizing their importance in maintaining vascular health [16].

### 2.2. Endothelial Functions Involving Integrins

Integrins are adhesive proteins essential for the functioning of the vascular endothelium [23]. They are transmembrane receptors that link the cytoskeleton with the extracellular matrix (ECM) and other cells, playing a diverse role in endothelial function [24]. As shown in Table 2., integrins mediate adhesion, migration, signal transduction, and cytoskeleton regulation, making them crucial for maintaining vascular homeostasis and for an adequate response to injury and inflammation [25].

### 2.3. Therapeutic Importance of Integrins in Vascular Disease

Understanding the role of integrins in the pathogenesis of vascular diseases has opened new therapeutic avenues, offering potential treatments for a wide range of conditions [46]. Research into integrin inhibitors-such as monoclonal antibodies and small molecules-has shown promising results in the treatment of cancers, inflammatory disorders, and cardiovascular diseases. For example, blocking integrins αVβ3 and αVβ5 can effectively inhibit tumor angiogenesis, thereby limiting tumor growth and metastasis [47].

Modulating integrin activity holds considerable therapeutic potential in managing vascular diseases. In atherosclerosis, integrin inhibitors may reduce leukocyte adhesion to vessel walls, thereby preventing plaque formation and progression [48]. Similarly, in hypertension, targeted therapies that affect integrin function can help prevent vascular remodeling, ultimately reducing vascular resistance and controlling blood pressure.

Beyond these roles in disease prevention, integrin modulation can also support tissue repair and regeneration [49]. Enhancing integrin activity can facilitate angiogenesis, which is crucial for tissue healing and regeneration, making integrins valuable targets in regenerative medicine and tissue engineering. Strategies that leverage integrins to promote new blood vessel formation and improve tissue repair could significantly enhance outcomes in patients requiring regenerative therapies.

The dual approach-targeting integrins to both inhibit pathological processes and promote repair mechanisms highlights the versatility of integrin-based therapies. Such treatments could not only alleviate vascular conditions but also play a key role in improving tissue health in broader therapeutic contexts, including wound healing, ischemic conditions, and cancer management [50].

## 3. Integrins as Key Adhesion Proteins

Integrins are fundamental receptors on the cell surface that mediate cell adhesion to ECM proteins and, in some cases, facilitate cell-cell adhesion [51,52]. As described in Table 3, these receptors are composed of two distinct subunits (α and β) that form heterodimers, resulting in diverse combinations and specificities. Integrins are crucial for regulating multiple biological processes, including adhesion, migration, signal transduction, and cellular differentiation. They play a vital role in maintaining vascular integrity, responding to injury, and ensuring proper immune system function. The following table summarizes the key structural and functional characteristics of integrins, highlighting their importance in cellular adhesion and signaling mechanisms.

### 3.1. Role of Integrins in the Vascular System

Integrins are pivotal in maintaining the proper functioning of the vascular system, particularly within endothelial cells that line blood vessels. They mediate adhesion to the ECM, regulate cell migration during angiogenesis, and contribute to vascular integrity. In different segments of the vascular system, such as capillaries, arterioles, or large arteries, specific integrins perform specialized functions [58]:

1. Capillaries: Integrins like αvβ3 are essential for endothelial migration, supporting the formation of new vessels during angiogenesis [59].
2. Arterioles: Integrins, including β1, are crucial for endothelial polarization and lumen formation, which is important for vessel stability [60].

### 3.2. Integrins in Adhesion, Cellular Migration, and Signaling

Integrins mediate cell adhesion through interactions with ECM ligands, such as fibronectin, laminin, collagen, and others. The integrin signaling mechanism is bidirectional, encompassing both “inside-out” and “outside-in” signaling:

1. Inside-out signaling is initiated by intracellular proteins, such as talin and kindlin, which enhance integrin affinity for ECM ligands [61].
2. Outside-in signaling occurs upon ligand binding, inducing conformational changes that trigger intracellular responses, including cytoskeletal rearrangement and activation of signaling pathways [62].

This bidirectional signaling facilitates the regulation of diverse cellular processes, including adhesion, migration, proliferation, and differentiation. Integrins can be grouped based on their ligand specificity into four categories: laminin receptors, Arginine-Glycine-Aspartic Acid (RGD) receptors, collagen receptors, and leukocyte-specific receptors [63]. Table 4. provides a classification of integrins based on their receptor specificity, distribution in different cell types, and the ligands they bind. Such classification helps understand their diverse roles in the vascular system and immune responses.

#### Crosstalk Between Integrin and Cytokine Signaling

The crosstalk between integrin and cytokine signaling represents a fundamental regulatory network that orchestrates cellular adhesion, migration, immune response, and tissue remodeling. This dynamic interaction allows cytokines to modulate integrin expression and activation while integrins reciprocally influence cytokine signaling pathways. Such bidirectional signaling ensures that cellular responses are finely tuned in response to environmental cues. This interplay plays a pivotal role in various physiological processes, including angiogenesis, immune cell trafficking, and tissue repair, as well as in pathological conditions such as atherosclerosis, cancer, and chronic inflammatory diseases [71].

The influence of cytokines on integrin expression is mediated through the activation of key transcription factors, including NF-κB, STAT3, and AP-1 [72,73,74]. Cytokines such as TNF-α, IL-1β, IL-6, and IFN-γ activate their respective receptors (e.g., TNFR, IL-1R, IL-6R) and initiate signaling cascades, including the JAK/STAT pathway [75], as well as MAPK and NF-κB pathways. These cascades collectively upregulate the transcription of genes encoding integrins such as α4, α5, β1, β2, and αvβ3. The increased surface expression of these integrins enhances cellular adhesive capacity, enabling immune cells, endothelial cells, and cancer cells to adhere to ECM components and cell surface ligands. For instance, in vascular endothelial cells, the induction of VCAM-1 and ICAM-1 by cytokines allows for strong interactions with β2 integrins on leukocytes, facilitating immune cell recruitment to sites of inflammation. Similarly, αvβ3 and α5β1 integrins play a central role in tumor cell invasion and endothelial cell migration during angiogenesis, processes that are heavily influenced by cytokine activity.

Integrins not only act as passive responders to cytokine signaling but also actively regulate cytokine production and signaling cascades through outside-in signaling. Upon engagement with ECM ligands such as fibronectin, collagen, and laminin, integrins undergo conformational changes, leading to their clustering and the recruitment of intracellular adapter proteins like talin, vinculin, and paxillin [53]. This process activates FAK (focal adhesion kinase) and Src kinases, which serve as molecular switches that trigger downstream signaling pathways, including the MAPK/ERK, NF-κB, and PI3K/AKT cascades. Activation of these pathways promotes not only cell survival, proliferation, and migration but also amplifies cytokine production [76]. The phosphorylation of IKK (IκB kinase) by integrin-activated FAK or cytokine-activated receptors results in the degradation of IκB, freeing NF-κB to translocate to the nucleus. Once in the nucleus, NF-κB induces the transcription of pro-inflammatory cytokines such as IL-6, TNF-α, and IL-1β, creating a positive feedback loop that reinforces the inflammatory response. This synergistic interaction between integrin and cytokine signaling ensures sustained immune cell activation and chronic inflammation [7].

The relationship between integrins and cytokines is also mediated by their physical interaction at the plasma membrane, where integrins form receptor complexes with cytokine receptors [11]. These complexes facilitate the cross-activation of signaling pathways, thereby amplifying cellular responses. For instance, the clustering of TNFR with β1 integrins enhances the sensitivity of cells to TNF-α, prolonging NF-κB activation and sustaining pro-inflammatory gene transcription. Similar interactions have been observed between IL-1R and β2 integrins, which enable endothelial cells and leukocytes to mount rapid inflammatory responses [77]. Such receptor crosstalk allows for greater specificity and responsiveness to environmental stimuli, providing a finely tuned system for cellular adaptation [78].

### 3.3. Structure, Activation, and Regulation of Integrins

Integrins are complex proteins with a dynamic structure that includes extracellular, transmembrane, and cytoplasmic domains:

1. Extracellular Domain: This domain is responsible for recognizing ligands and is composed of the α subunit’s seven-bladed β-propeller, thigh region, and calf domains, along with the β subunit’s seven distinct domains [79].
2. Transmembrane Domain: Spans the cell membrane, connecting the extracellular and intracellular environments, and serves as a conduit for external signals to be transmitted into the cell [80].
3. Cytoplasmic Domain: Interacts with the cytoskeleton through intermediary proteins such as talin and kindlin. This interaction is vital for integrin activation and conformational changes necessary for ligand binding [81].

Integrins are not constitutively active. Their activation involves complex regulation. Cytoplasmic proteins like talin and kindlin bind to integrins and induce conformational changes from a bent, inactive state to an extended, active form. This activation is crucial for ligand binding and subsequent cellular responses, such as migration and survival [82]. 

In addition to affinity regulation, integrin-mediated adhesion is significantly influenced by avidity modulation. This process involves the dynamic regulation of the clustering and spatial organization of integrins on the cell surface, which collectively enhances the overall adhesive strength of the cell. Unlike affinity modulation, which alters the conformational state of individual integrins to increase ligand-binding capacity, avidity modulation focuses on the cooperative interactions of multiple integrins. This mechanism is crucial in ensuring robust and adaptive adhesion to the ECM or other cells, particularly in dynamic environments [83,84].

Avidity modulation is orchestrated by intracellular signaling pathways that reorganize the cytoskeleton and facilitate integrin clustering within the plasma membrane. Key adapter proteins such as talin, kindlin, and paxillin mediate this clustering, enhancing ligand-binding site density and stabilizing cell-ECM interactions. This increased integrin density amplifies outside-in signaling by promoting the formation of focal adhesion complexes, which serve as hubs for downstream signaling cascades, including FAK/Src and MAPK/ERK pathways. These pathways regulate essential cellular processes such as migration, proliferation, and survival [27,85,86].

In the vascular system, avidity modulation enables endothelial cells to adapt to mechanical and chemical stimuli, ensuring effective adhesion and migration during angiogenesis and vascular repair. Similarly, leukocytes rely on this mechanism to transition from rolling to firm adhesion on the endothelium, a critical step in immune cell trafficking. Dysregulation of avidity modulation is implicated in pathological conditions, including enhanced tumor cell invasiveness in cancer and aberrant leukocyte adhesion in atherosclerosis [83,84,86].

By integrating spatial organization and signaling dynamics, avidity modulation complements affinity changes, providing a versatile and robust mechanism for fine-tuning integrin function in both physiological and pathological contexts [83,85,86].

Allosteric activation of integrins is a fundamental mechanism regulating their adhesive function and cellular responses. This process enables integrins to dynamically switch between inactive, intermediate, and active conformations, allowing them to control cell adhesion, migration, and signaling [87]. Given the importance of integrins in numerous physiological and pathological processes, understanding the allosteric mechanism opens new possibilities for therapeutic intervention [88].

**Figure 1:**
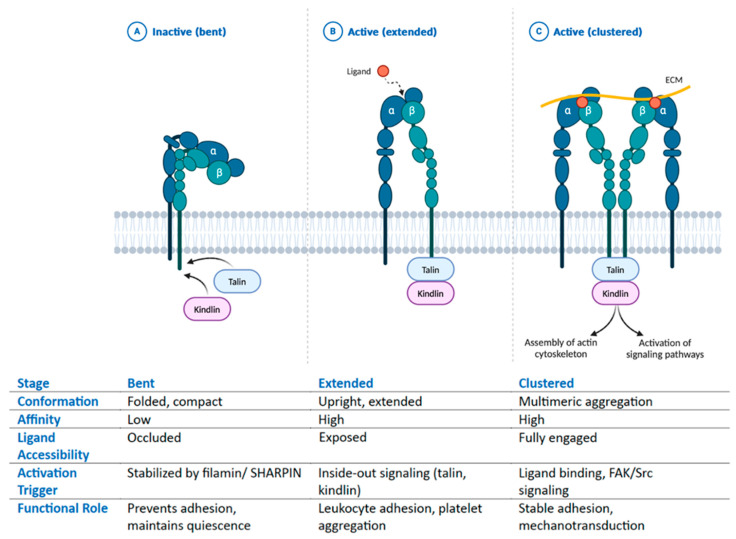
Integrin Activation States and Functional Roles. This figure illustrates the three major conformational states of integrins: inactive (bent), active (extended), and clustered (active), highlighting their structural features, ligand accessibility, activation triggers, and functions. (**A**) Inactive (bent): Integrins adopt a folded, compact conformation with low ligand affinity and occluded binding sites. Stabilized by filamin and SHARPIN, this state prevents adhesion and maintains cellular quiescence. (**B**) Active (extended): Upon inside-out signaling (talin, kindlin), integrins shift to an upright, extended structure, increasing ligand affinity and accessibility. This enables leukocyte adhesion and platelet aggregation, which are essential for immune responses and hemostasis. (**C**) Active (clustered): Integrins form multimeric aggregates, enhancing ligand engagement and intracellular signaling. Activation via ligand binding and FAK/Src signaling promotes stable adhesion and mechanotransduction, supporting cytoskeleton assembly and signal transduction.

As mentioned in Figure 1. integrins exist in three main conformational states: bent, extended and clustered.

The bent conformation is the default, low-affinity state in which the integrin remains folded, with its headpiece positioned close to the plasma membrane. In this state, the α and β subunits are tightly associated at their cytoplasmic tails, and their extracellular segments are bent, preventing ligand access to the binding site [53]. This conformation is stabilized by inhibitory proteins such as filamin and SHARPIN, which block the interaction of the β-subunit tail with activating proteins like talin and kindlin. The transmembrane helices of the α and β subunits are closely packed, maintaining the bent posture [27]. The metal-ion-dependent adhesion site (MIDAS), which is essential for ligand binding, is occluded in this conformation, rendering the integrin inactive [89,90]. This bent state ensures that integrins remain in a non-adhesive mode, which is crucial for preventing unnecessary adhesion of leukocytes to endothelial cells or platelets to each other in the absence of injury or inflammation. It also allows cells to maintain a “standby” state, ready to respond to activation signals as needed.

The extended, ligand-ready state occurs when intracellular signals trigger a conformational change via a process known as inside-out signaling [91,92]. Signals from G-protein-coupled receptors (GPCRs) or receptor tyrosine kinases (RTKs) recruit and activate intracellular proteins such as talin and kindlin [93]. Talin binds to the cytoplasmic tail of the β-subunit, inducing the separation of the α and β subunit tails. This causes the transmembrane helices to separate, destabilizing the compact structure and straightening the extracellular headpiece. As a result, the headpiece of the integrin extends outward from the plasma membrane, exposing the ligand-binding site. Kindlin works in concert with talin, stabilizing the conformational change and ensuring the high-affinity ligand-binding state [94]. This shift increases the affinity for extracellular ligands like fibronectin, laminin, and collagen. The MIDAS site becomes exposed and ready to coordinate divalent cations (Mg²⁺ or Mn²⁺), further stabilizing ligand binding [95]. This extended conformation is essential for processes like leukocyte adhesion, platelet aggregation, and endothelial migration, where integrins must bind strongly to ECM components or endothelial adhesion molecules such as ICAM-1 and VCAM-1. Without this transformation, immune cells could not adhere to inflamed endothelium, nor could platelets bind fibrinogen during clot formation [96].

Finally, the clustered state represents the most stable and active conformation of integrins [97,98]. This state occurs following ligand binding and is a result of outside-in signaling, where ligand binding triggers the clustering of multiple integrins within the membrane, forming high-density focal adhesion complexes [83]. Ligand binding stabilizes the extended conformation, locking it into place. Clustering is driven by lateral diffusion of integrins within the plasma membrane and by intracellular forces exerted through proteins like talin, vinculin, and paxillin [99]. This clustering increases the strength of the interaction between the cell and the ECM. Once clustering is initiated, focal adhesion kinase (FAK) and Src kinases are activated, leading to the phosphorylation of downstream signaling proteins like talin, vinculin, and paxillin [100]. These events facilitate the assembly of focal adhesion complexes, which serve as hubs for cytoskeletal anchoring and intracellular signaling. The activation of signaling cascades, such as the FAK/Src, MAPK/ERK, and PI3K/AKT pathways, promotes cell survival, migration, and mechanotransduction. This state is essential for stable adhesion, mechanosensing, and force transmission during processes like angiogenesis, immune cell extravasation, and tissue repair [101]. Clustering of integrins enables cells to form stable, long-term adhesions with the ECM, allowing endothelial cells, fibroblasts, and cancer cells to migrate and invade tissues [102].

## 4. Functions of Integrins in Different Vascular Segments

Integrins play diverse roles in different types of blood vessels, adapting their functions to the specific mechanical and functional requirements of each segment of the vascular system. Through interactions with ligands, integrins regulate vascular function, immune responses, angiogenesis, and lymphatic vessel development [103]. Dysregulation of integrin signaling pathways can lead to vascular dysfunction and contribute to the onset of vascular diseases.

Understanding the specific roles of integrins in different vascular compartments is critical for developing targeted therapeutic strategies for vascular-related disorders [104]. Each segment of the vascular system has distinct functions regulated by particular integrins, influencing cell adhesion, migration, and responses to mechanical and inflammatory stimuli, as summarized in Table 5.

### 4.1. Capillaries 

In capillaries, integrins such as αvβ3 and α5β1 play crucial roles in regulating endothelial adhesion, migration, and angiogenesis [116]. Integrin αvβ3 is particularly important for the migration of endothelial cells and the formation of new blood vessels, while α5β1 supports the differentiation of endothelial progenitor cells, essential for capillary maintenance.

In addition to promoting angiogenesis, integrins regulate vessel permeability and mediate interactions between endothelial cells and immune cells, facilitating leukocyte adhesion during inflammation. These combined functions help maintain proper capillary health in both normal and inflammatory conditions [117].

Role in disease progression: Dysregulation of integrins in capillaries can lead to impaired angiogenesis, reduced vessel integrity, and altered inflammatory responses, contributing to chronic inflammation or insufficient tissue repair.

Hodivala-Dilke and her team focused on the versatile role of integrins in angiogenesis, emphasizing their complexity. Analyzing αvβ3, the authors highlighted this integrin’s ability to bind to a wide range of molecules, including vitronectin, fibronectin, thrombospondin, MMP2, and various collagen fragments. A key conclusion from their research is that these interactions can either promote or inhibit angiogenesis, depending on the ligands involved and the cellular context.

Hodivala-Dilke et al. also observed that mice lacking β3 exhibit increased angiogenesis compared to wild-type mice, suggesting that αvβ3 may play a role in limiting angiogenesis under certain conditions. They proposed that the presence of unbound αvβ3 may induce endothelial cell apoptosis, leading to the suppression of angiogenesis [118].

Laurens et al. focused on the interdependence of αvβ3 and α5β1 in the formation of capillary structures within a three-dimensional fibrin matrix. In their in vitro studies, using human microvascular endothelial cells (hMVEC) cultured in a fibrin matrix, Laurens and her team found that blocking αvβ3 or α5β1 individually did not significantly reduce tube formation. Notably, only the simultaneous inhibition of both integrins effectively suppressed this process, highlighting their synergistic role in angiogenesis. The authors suggested that this synergy might stem from αvβ3 and α5β1 binding to different sites on fibrin, allowing them to jointly regulate adhesion, migration, and tube formation by endothelial cells [119]. 

Hodivala-Dilke’s and Laurens’ teams made significant contributions to understanding the roles of αvβ3 and α5β1 integrins in angiogenesis. Hodivala-Dilke highlighted the complexity of αvβ3’s functions, pointing to its ability to both promote and inhibit angiogenesis depending on the context. In contrast, Laurens focused on the synergistic action of αvβ3 and α5β1, discovering that their simultaneous blockade is essential for effectively inhibiting blood vessel formation within a fibrin matrix.

### 4.2. Arterioles

In arterioles, β1 integrins are essential for endothelial cell polarization and lumen formation, ensuring that these small vessels maintain proper structure and function. β1 integrin also stabilizes cell-cell junctions and regulates endothelial alignment, which is crucial for blood flow regulation [120].

Role in disease progression: Dysfunction in β1 integrin can lead to improper vascular lumen formation, reduced blood flow efficiency, and increased susceptibility to vascular disorders.

Orlando deLeon et al. describe the impact of β1 integrin on polarization and lumen formation in three-dimensional cultures of MDCK cells. β1 integrin, as a cell-ECM adhesion receptor, plays a crucial role in the correct orientation of the apical domain in epithelial cells. Its inhibition leads to reversed polarization and blocked lumen formation. This occurs because β1 integrin activates the Rac1 signaling pathway, which in turn inhibits RhoA and ROCK1 activity, thereby reducing actomyosin contractility. Reduced contractility is essential for the proper formation of a laminin-rich basement membrane. The basement membrane, in turn, is critical for proper apical domain orientation and lumen formation. Inhibition of β1 integrin function results in increased RhoA activity and, consequently, excessive actomyosin contractility. This disrupts basement membrane formation, leading to defects in polarization and lumen formation. Orlando deLeon et al. emphasize that constitutively active Pak1, a Rac1 effector, mimics the phenotype induced by β1 integrin inhibition. This suggests that Pak1 may indirectly influence β1 integrin functions by regulating ECM adhesion dynamics and basement membrane formation [121].

### 4.3. Arteries

Integrins α1β1 and α2β1 in arteries are responsible for stabilizing the arterial walls by interacting with ECM components such as collagen and elastin [122]. This stabilization is critical for maintaining the mechanical strength of arteries, which must withstand high pressure and pulsatile blood flow. Integrins also help regulate smooth muscle cell contraction and relaxation, thereby contributing to blood pressure control [123].

During arterial injury or inflammation, integrins mediate immune cell adhesion and migration, facilitating tissue repair and controlling inflammation [124].

Role in disease progression: Impaired integrin function can lead to weakened arterial walls, making them more prone to damage, aneurysms, or atherosclerosis.

Finney et al. discuss the role of integrins in the development of atherosclerosis. They emphasize that integrins influence many aspects of this condition, from the early stages of inflammation to the progression of advanced atherosclerotic plaques. Integrins regulate endothelial phenotype, facilitate leukocyte migration, impact their functions, and drive smooth muscle remodeling. Additionally, integrin signaling in platelets contributes to thrombotic complications, which are typically responsible for the clinical manifestation of cardiovascular diseases. The authors highlight that the microvascular environment plays a key role in the localization of atherosclerotic plaques and the sensitivity of plaque cells to various atherogenic stimuli. Atherosclerotic plaques develop in arterial regions exposed to turbulent blood flow patterns, while vascular areas subjected to unidirectional laminar flow are protected against plaque formation. Changes in the arterial matrix composition also regulate many aspects of plaque formation, including lipid deposition, inflammation, and smooth muscle incorporation [9].

On the other hand, Zeltz and Gullberg focus on the role of integrins in wound healing, fibrosis, and cancer. The authors suggest that collagen-binding integrins have a limited role in static tissue homeostasis but appear to be more important in dynamic events occurring during tissue injury, regeneration, and inflammation. Studies indicate that these integrins could serve as valuable biomarkers and prognostic and/or therapeutic targets in diseases. The article discusses various integrin types, including α1β1, α2β1, α10β1, and α11β1, and their specific roles in different processes. For example, integrin α1β1 regulates kidney homeostasis, and its absence exacerbates glomerulosclerosis following glomerular injury. Integrin α2β1 plays a role in hemostasis and wound healing, with its absence delaying fibrosis and glomerular damage. Integrin α10β1 is crucial for skeletal development, and its deficiency leads to chondrodysplasia. Meanwhile, integrin α11β1 participates in collagen remodeling, and its absence reduces wound tensile strength [125].

### 4.4. Veins

Integrins play a crucial role in maintaining endothelial cell integrity, regulating vascular tone, and responding to mechanical stress within the venous system. Specifically, integrins such as αvβ3 and α5β1 are essential for endothelial adhesion and migration, ensuring the structural stability and function of vein walls. By forming and maintaining adherens junctions, these integrins contribute to the integrity of the venous endothelium, which is critical for proper venous function [119].

In veins, where blood flow is slower, integrins help maintain a balance between anticoagulant and procoagulant properties of the vessel walls, thereby preventing excessive clotting and reducing the risk of thrombosis [126]. This regulation is particularly important in larger veins, where higher pressure necessitates mechanisms to preserve wall integrity and elasticity. Integrins like αvβ3 and α5β1 play a vital role in maintaining this balance, supporting overall vein durability and function [127].

Integrins are also involved in mechanotransduction, enabling endothelial cells to adapt to changes in blood flow and pressure by converting mechanical stimuli into biochemical signals. This ability helps the venous endothelium adjust to mechanical stress and maintain homeostasis under varying flow conditions.

Additionally, similar to their role in arteries, integrins in veins are crucial for mediating inflammatory responses by recruiting leukocytes to the endothelium. For example, integrins such as αLβ2 Lymphocyte Function-Associated Antigen 1 (LFA-1) facilitate the adhesion and migration of leukocytes to sites of inflammation or injury, which is essential for the immune response and tissue repair [128]. This mechanism helps control inflammation and supports the proper healing of venous tissues.

In summary, integrins in veins are involved in maintaining endothelial integrity, regulating coagulation, adapting to mechanical stress, and supporting immune responses, which are vital for venous health and function [129].

### 4.5. Lymphatic Vessels

In lymphatic vessels, integrins are essential for lymphangiogenesis, maintaining lymphatic endothelial cell integrity, and regulating lymph flow. Integrins such as α9β1 and α4β1 are particularly involved in the development and function of lymphatic vessels. Integrin α9β1, for instance, is crucial for the migration and proliferation of lymphatic endothelial cells, enabling the formation of new lymphatic vessels during both development and in response to injury [104].

Besides promoting lymphangiogenesis, integrins help maintain the structural integrity of lymphatic endothelial cells, which ensures the effective transport of lymph and immune substances [130]. They regulate lymph flow by modulating endothelial cell contractility and organization, allowing efficient response to changes in fluid dynamics and pressure, thus ensuring proper lymph circulation and immune function.

The team led by Koyu Ito and collaborators discovered that integrin α9β1 and its ligand, tenascin-C (TN-C), play a crucial role in regulating lymphocyte egress from draining lymph nodes (dLNs) during inflammation. Blocking α9 integrin signaling, either through the antibody 55A2C or the absence of TN-C in knockout mice, resulted in empty lymphatic sinuses in the dLNs, indicating impaired lymphocyte egress. Further studies demonstrated that stimulation of lymphatic endothelial cells (LECs) by TN-C increased the secretion of sphingosine-1-phosphate (S1P), a key factor in regulating lymphocyte egress. This mechanism likely does not affect the synthesis or degradation of S1P but rather its secretion, potentially through the regulation of an S1P transporter. Interestingly, α9 integrin blockade did not affect lymphangiogenesis induced by Freund’s adjuvant (CFA) or lymph flow, suggesting its role is focused on lymphocyte egress regulation rather than lymphatic vessel development [131].

In the experimental autoimmune encephalomyelitis (EAE) model, prophylactic administration of 55A2C alleviated disease symptoms and reduced inflammatory cell infiltration and demyelination in the spinal cord. This suggests that α9 integrin could be a potential therapeutic target in immune-mediated diseases by regulating lymphocyte egress from inflamed sites. The study also revealed that the activation of the β1 integrin subunit, the partner of α9 in forming α9β1 integrin, was increased in inflamed dLNs, indicating that the TN-C and α9β1 interaction occurs primarily in these nodes.

On the other hand, the research by the team of Nicholas E. Vlahakis et al. highlights the significant role of α9β1 in VEGF-A-induced angiogenesis. This integrin not only directly binds to VEGF-A but also cooperates with VEGF-R2, participates in signal transduction, and is essential for angiogenesis in vivo [132].

## 5. Integrins in Cardiovascular Diseases

Integrins play crucial roles in the maintenance of cardiovascular health through their regulation of endothelial function, vascular remodeling, and immune cell interactions. However, dysregulation in integrin expression or function can contribute to the onset and progression of various cardiovascular diseases. In this chapter, we discuss the role of integrins in atherosclerosis, hypertension, and chronic heart failure, as well as their relationship with inflammatory markers and examples of clinical studies that target integrins in these conditions.

### 5.1. Integrin Dysfunction in Atherosclerosis

Atherosclerosis is characterized by the buildup of lipid plaques within the arterial walls, leading to the narrowing of the arteries and impaired blood flow. Integrins play a pivotal role in the progression of atherosclerosis, primarily through their involvement in endothelial cell adhesion, immune cell recruitment, and vascular smooth muscle cell migration [133].

In the early stages of atherosclerosis, integrins such as α4β1 and αLβ2 facilitate the adhesion of leukocytes to the endothelial surface. These integrins interact with vascular cell adhesion molecule 1 (VCAM-1) and intercellular adhesion molecule 1 (ICAM-1), leading to leukocyte migration into the intima, where they contribute to inflammation and plaque development. Additionally, β1 and β3 integrins are involved in vascular smooth muscle cell migration and proliferation, further contributing to plaque formation and arterial remodeling [134].

Pathological Impact: Dysregulated integrin signaling can enhance the recruitment of inflammatory cells and promote smooth muscle cell proliferation, accelerating plaque buildup and increasing the risk of plaque rupture, which may result in acute cardiovascular events such as myocardial infarction or stroke [135].

### 5.2. Changes in Integrin Expression and Function in Hypertension

Hypertension is associated with alterations in vascular structure, including increased stiffness and remodeling of blood vessels. Integrins contribute to these processes by regulating vascular smooth muscle cell behavior and ECM interactions.

Integrins such as α5β1 and αvβ3 are involved in the remodeling of the arterial wall by promoting the adhesion and migration of smooth muscle cells [136]. This contributes to increased vessel stiffness and elevated vascular resistance, which are hallmarks of hypertension. Furthermore, β1 integrins facilitate the deposition of ECM components such as collagen, leading to fibrosis and increased arterial stiffness.

Pathological Impact: Overexpression or hyperactivation of integrins in hypertension can exacerbate vascular remodeling, leading to an increase in peripheral resistance and sustained high blood pressure, which places additional strain on the heart and increases the risk of end-organ damage.

### 5.3. Role of Integrins in Chronic Heart Failure

Chronic heart failure (CHF) is characterized by the heart’s inability to pump blood effectively, often resulting from sustained hypertension or myocardial injury [137]. In CHF, integrins are implicated in both the remodeling of cardiac tissue and the regulation of inflammatory responses.

Integrins such as α7β1 and β1 are important for maintaining the integrity of cardiomyocytes and their attachment to the ECM [138]. In heart failure, changes in integrin expression lead to impaired cardiomyocyte-ECM interactions, contributing to cardiomyocyte apoptosis and fibrosis. Additionally, αvβ3 integrins are involved in promoting angiogenesis, which is often impaired in the failing heart, leading to inadequate blood supply and further functional decline.

Pathological Impact: Dysfunctional integrin signaling in CHF can lead to an increase in myocardial fibrosis, reduced contractility, and impaired adaptation to hemodynamic stress, which collectively exacerbate heart failure progression.

## 6. Integrin-Cytokine Interactions and Their Clinical Implications

Integrins are pivotal mediators of cell-ECM interactions and are closely associated with inflammatory markers, playing a critical role in processes such as cardiovascular diseases, autoimmune disorders, and cancer. Cytokines, including TNF-α and IL-6, upregulate adhesion molecules like ICAM-1 and VCAM-1, enhancing integrin-mediated leukocyte adhesion to the endothelium [139]. This mechanism intensifies the inflammatory response within the vascular wall, contributing to vascular damage, plaque instability in atherosclerosis, and vascular remodeling in hypertension [140].

Selectins (P-selectin and E-selectin) complement integrin function by mediating leukocyte rolling, firm adhesion, and transmigration into inflamed tissues [141]. This coordinated interplay amplifies local inflammatory responses, emphasizing the critical role of integrins and cytokines in driving chronic inflammation.

### 6.1. Interaction of Integrins with Inflammatory Cytokines

Cytokines regulate integrin expression, clustering, and activation of various cell types, including leukocytes, endothelial cells, and fibroblasts. This regulation involves transcriptional activation and protein trafficking mediated by pathways such as NF-κB, JAK/STAT, and AP-1, which promote the transcription of integrin subunit genes (e.g., α4, β1, β2) [142]. As a result, integrins such as α4β1 (VLA-4) and αMβ2 (Mac-1) are expressed on leukocytes, facilitating adhesion and transmigration through ICAM-1 and VCAM-1-enriched endothelial barriers [142].

Cytokines also modulate integrin activation via inside-out signaling, involving adapter proteins like talin and kindlin. These proteins bind to integrin cytoplasmic tails, inducing conformational changes that enhance ligand affinity. Additionally, cytokines can directly interact with integrins, as IL-1β binds β2 integrins to stabilize ICAM-1 interaction, and TNF-α binds αvβ3 and α5β1 integrins, triggering outside-in signaling [143].

### 6.2. Signaling Pathways 

The interplay between integrins and cytokines activates several signaling pathways critical to inflammation and tissue remodeling:

FAK/Src Pathway: Integrin engagement induces FAK autophosphorylation, recruiting Src kinases and forming focal adhesion complexes with paxillin, vinculin, and talin. These complexes stabilize integrin-ECM interactions and regulate cytoskeletal reorganization, supporting angiogenesis, cancer metastasis, and endothelial repair [144].

NF-κB Pathway: Integrin and cytokine receptor activation triggers phosphorylation of IKK, leading to IκB degradation and NF-κB nuclear translocation [145]. 

NF-κB drives the expression of pro-inflammatory cytokines (TNF-α, IL-6, IL-1β) and adhesion molecules (ICAM-1, VCAM-1), sustaining chronic inflammation [140,146].

MAPK/ERK Pathway: FAK/Src signaling activates the Ras-Raf-MEK-ERK axis, enhancing endothelial migration, tissue remodeling, and tumor invasion [147].

PI3K/AKT Pathway: Integrin or cytokine receptor activation recruits PI3K, generating PIP3 and activating AKT. This pathway enhances cell survival, proliferation, and cytokine secretion, further amplifying inflammation [148,149,150].

### 6.3. Clinical Implications

The integrin-cytokine crosstalk has profound clinical implications in various diseases, especially in the context of cardiovascular diseases, autoimmune disorders, and cancer [151]. Cytokine-induced upregulation of VCAM-1 and ICAM-1 promotes leukocyte infiltration and plaque development in atherosclerosis. Targeting β2 integrins or NF-κB signaling has been shown to reduce vascular inflammation and disease progression [152].

In autoimmune diseases such as multiple sclerosis, the α4β1-VCAM-1 axis enables leukocytes to cross the blood-brain barrier, facilitating the infiltration of autoreactive immune cells into the central nervous system [153]. The monoclonal antibody natalizumab blocks α4 integrins, preventing immune cell infiltration and reducing disease progression [154].This therapeutic strategy underscores the importance of blocking the integrin-cytokine interface in autoimmune pathologies [142].

In cancer, the combined action of cytokines and integrins drives tumor growth, metastasis, and angiogenesis [148]. Cytokines like IL-6 and IL-1β enhance the expression of αvβ3 and α5β1 integrins on tumor cells, while integrin engagement activates the FAK/Src, MAPK/ERK, and PI3K/AKT pathways, promoting cancer cell survival, migration, and resistance to therapy [43]. Anti-integrin therapies like cilengitide (targeting αvβ3/αvβ5) are being explored as anti-metastatic agents [59]. Together, the clinical relevance of integrin-cytokine crosstalk highlights the potential of targeting this interface as a therapeutic strategy for chronic inflammation, cardiovascular disease, and cancer [155].

## 7. Targeted Therapies for Integrins

Integrins have become attractive therapeutic targets due to their pivotal role in regulating cellular adhesion, migration, and signaling in the vascular system. Targeted therapies that modulate integrin activity are being explored for the treatment of various cardiovascular diseases and inflammatory conditions, as well as regenerative medicine. This chapter presents different approaches, including integrin inhibitors, monoclonal antibodies, gene therapy, and pharmacological modulators, along with recent scientific findings and their potential therapeutic implications.

### 7.1. Integrin Inhibitors in Treating Vascular Diseases

Integrin inhibitors represent a promising class of therapeutics in the treatment of vascular diseases [53]. These inhibitors can specifically block integrin-ligand interactions, reducing pathogenic processes like inflammation, thrombosis, and tissue remodeling [152]. Recent clinical trials and experimental studies have investigated various inhibitors with potential therapeutic effects:

Cilengitide is a well-studied cyclic peptide that inhibits αvβ3 and αvβ5 integrins, initially developed for oncology [156].Cilengitide has shown potential in inhibiting neovascularization in atherosclerotic plaques, thereby reducing the risk of plaque rupture and cardiovascular events [157].

A study by Meester et al. demonstrated that cilengitide can reduce the progression of plaque formation and improve endothelial function in experimental models, highlighting its potential to stabilize vulnerable plaques and reduce the risk of cardiovascular events [152,158].

Tirofiban and eptifibatide are GPIIb/IIIa inhibitors (an integrin found on platelets) used to prevent thrombosis in patients with acute coronary syndrome [159]. 

Their effectiveness in preventing platelet aggregation has been demonstrated in multiple clinical studies [160,161,162].

### 7.2. Monoclonal Antibodies Against Integrins

Monoclonal antibodies targeting integrins offer another approach for controlling integrin-mediated pathways, providing specificity in modulating integrin activity in different disease contexts. These antibodies are engineered to selectively bind and inhibit integrins involved in pathogenic processes:

Natalizumab is a monoclonal antibody that targets α4 integrins and has been widely used to treat autoimmune diseases like multiple sclerosis (MS) [163].

Its role in vascular diseases, particularly in preventing leukocyte recruitment to inflamed vascular tissues, has been explored. In the Morrow et al. study, natalizumab was found to be highly effective in reducing relapse rates and controlling disease progression in patients with relapsing-remitting multiple sclerosis. The study particularly highlighted the benefits of extended interval dosing, which helped reduce the risk of progressive multifocal leukoencephalopathy (PML) without significantly compromising treatment efficacy. This approach provided a safer, long-term management strategy for MS patients at higher risk of PML, balancing efficacy and safety [164].

Etaracizumab targets αvβ3 integrins and has been studied for its anti-angiogenic effects, which are critical in reducing plaque vulnerability in atherosclerosis [136]. The study by Delbaldo et al. concluded that etaracizumab is a promising therapeutic candidate targeting angiogenesis and could be particularly useful in solid tumors reliant on neovascularization for growth [165].

However, the clinical development of etaracizumab was halted despite its potential, reflecting the challenges in translating promising preclinical findings into sustained therapeutic success [166].

### 7.3. Modulation of Integrin Activity in Regenerative Therapy and Tissue Engineering

Modulating integrin activity has shown considerable promise in enhancing tissue regeneration and supporting tissue engineering approaches. By promoting integrin-mediated cell adhesion, migration, and differentiation, regenerative therapies aim to improve tissue repair following injury.

RGD-functionalized biomaterials have been developed to mimic integrin-binding motifs, promoting cell adhesion and tissue regeneration [167].A study by Huang et al. used RGD-functionalized polyglycolic acid (PGA) materials to show enhanced endothelial cell adhesion and spreading, which is crucial for the regeneration of vascular tissues [168]. Another study by Yang et al. highlights how RGD-modified alginate scaffolds enhanced vascular tissue regeneration, improving cell morphology and collagen deposition [169].

Hydrogels functionalized with α5β1 integrin-binding motifs have also been used in tissue engineering to promote endothelial cell adhesion and angiogenesis. In the study by Shuoran Li et al., the researchers explored how the activation of specific integrins can influence vascular formation and permeability in engineered hydrogel scaffolds. They demonstrated that promoting α3/α5β1 integrin binding leads to the formation of mature and non-leaky blood vessels, both in vitro and in vivo [170]. This contrasts with αvβ3 integrin binding, which resulted in disorganized, clumped endothelial sprouts and leaky vessels.

### 7.4. Gene Therapies and Their Potential in Modulating Integrin Function

Gene therapy represents a cutting-edge approach to modulating integrin expression and function, offering opportunities to correct dysregulated integrin pathways at the genetic level. By using gene-editing technologies such as clustered regularly interspaced short palindromic repeats-associated protein 9 (CRISPR/Cas9), researchers have been able to selectively upregulate or downregulate integrin genes to improve cardiovascular health.

CRISPR/Cas9-mediated integrin gene editing has been investigated in experimental models of atherosclerosis [78]. A study by Chen demonstrated that CRISPR/Cas9 can be a powerful tool not only in cancer immunotherapy but also in the regulation of immune responses in cardiovascular diseases, especially those linked with obesity, inflammation, and atherosclerosis [78,171].

Adeno-associated viruses (AAVs) are frequently employed in gene therapy due to their safety and ability to target a wide variety of tissues, including cardiac tissue. Research has shown that AAV-mediated delivery of therapeutic genes can lead to improved cardiomyocyte survival and reduced fibrosis, which are critical for treating chronic heart failure. These vectors have been optimized to efficiently deliver genes to the heart, helping enhance integrin expression and, consequently, cardiac repair [172,173].

### 7.5. Pharmacological Modulators of Integrins

Pharmacological modulators of integrins include small molecules designed to either inhibit or activate integrins in a disease-specific context, offering flexibility in integrin-targeted therapy.

Existing studies on ATN-161, a small peptide that inhibits integrins like α5β1 and αvβ3, show its potential as a therapeutic agent in cardiovascular diseases, particularly in reducing plaque formation and improving endothelial function. A 2021 study explored the impact of ATN-161 in models of atherosclerosis. It demonstrated that treatment with ATN-161 reduced plaque size and enhanced endothelial function in animal models, such as rabbits fed a high-cholesterol diet. This suggests that inhibiting integrins with ATN-161 can decrease inflammation and fibrosis, crucial factors in plaque stability and vascular health [174,175].

Pirfenidone has shown promise as an antifibrotic agent in treating heart failure with preserved ejection fraction (HFpEF) and myocardial fibrosis, as evidenced in recent clinical research. A key study, the PIROUETTE trial, explored the effects of pirfenidone in patients with HFpEF and confirmed its role in reducing myocardial fibrosis. Pirfenidone works by inhibiting the transforming growth factor β (TGF-β1) pathway, which is central to the development of fibrosis, and it reduces the activity of β1 integrins, which are involved in cardiac tissue remodeling. Patients treated with pirfenidone demonstrated improvements in cardiac function, reduced fibrosis, and better overall heart health [176,177,178].

## 8. Summary

Dysfunction of integrins is a significant contributing factor to the development of various cardiovascular diseases. In atherosclerosis, integrin-mediated leukocyte adhesion and smooth muscle cell migration contribute to plaque formation and instability. In hypertension, dysregulated integrin signaling enhances vascular remodeling, leading to increased arterial stiffness and peripheral resistance. In chronic heart failure, altered integrin expression and signaling pathways contribute to cardiomyocyte apoptosis and myocardial fibrosis, exacerbating disease progression. Thus, understanding the mechanisms underlying integrin dysfunction is vital for elucidating the pathogenesis of cardiovascular diseases.

With advancements in understanding integrin signaling mechanisms, new therapeutic opportunities have emerged. Targeting integrins with inhibitors, monoclonal antibodies, and gene therapies presents significant potential in treating cardiovascular diseases. Integrin inhibitors, such as cilengitide and GPIIb/IIIa inhibitors, have shown promise in reducing plaque progression and preventing thrombotic events. Monoclonal antibodies targeting specific integrins have demonstrated efficacy in controlling inflammation and vascular remodeling. Moreover, novel approaches involving gene therapy and pharmacological modulators have been developed to selectively regulate integrin function, offering personalized treatment options. In regenerative medicine, integrin-targeted biomaterials and hydrogels enhance tissue repair, providing new possibilities for tissue engineering and vascular regeneration.

Future research on integrins in the context of cardiovascular diseases is poised to focus on several key areas:

1. Development of Specific and Selective Integrin Modulators: Given the widespread expression of integrins across different tissues, achieving specificity in targeting integrin-mediated pathways remains a challenge. Future studies should aim at developing more selective inhibitors and antibodies to minimize off-target effects and maximize therapeutic efficacy.
2. Personalized Therapies Based on Integrin Profiling: Advances in genomic and proteomic technologies could enable integrin profiling in patients with cardiovascular diseases. Identifying specific integrin expression patterns could help tailor therapies to individual patients, enhancing treatment outcomes and reducing adverse effects.
3. Regenerative and Reparative Applications: Research should continue to explore the role of integrins in tissue regeneration and repair, particularly in the context of heart failure and vascular injury. Leveraging integrin-modulating biomaterials and gene editing techniques, such as CRISPR, could enhance tissue repair capabilities and improve outcomes in patients with significant cardiovascular damage.
4. Integrin Interaction Networks: Understanding how integrins interact with other molecules, such as cytokines, growth factors, and other adhesion receptors, could reveal new therapeutic targets and offer insights into the complex network of signaling pathways involved in cardiovascular health and disease.
5. Clinical Translation: Continued efforts are needed to translate findings from preclinical studies into clinical applications. Large-scale clinical trials will be crucial for evaluating the safety and efficacy of new integrin-targeting therapies and determining their place in standard treatment regimens for cardiovascular diseases.

In conclusion, integrins are key mediators of cell adhesion, migration, and signaling in the cardiovascular system. Their dysfunction is intricately linked to the progression of cardiovascular diseases, making them valuable targets for innovative therapeutic approaches. The future of integrin research holds promise for developing more specific, effective, and personalized therapies, ultimately improving the outcomes for patients suffering from cardiovascular conditions.

## 9. Conclusions

In conclusion, integrins are central regulators of cardiovascular health, orchestrating endothelial adhesion, migration, and signaling pathways critical for vascular homeostasis. Dysregulation of integrin function is a key driver in the pathogenesis of atherosclerosis, hypertension, and heart failure, contributing to inflammation, vascular remodeling, and plaque instability.

This review explores the transformative potential of integrin-targeted therapies, including inhibitors, monoclonal antibodies, and gene-editing technologies, in mitigating pathological processes and promoting vascular repair. Innovations such as integrin-modulating biomaterials further expand the frontier of regenerative medicine, offering new opportunities for tissue repair and angiogenesis.

Future directions should prioritize the development of highly specific integrin modulators, unravel the complexity of integrin signaling networks, and accelerate the translation of these advances into clinical applications, ultimately redefining therapeutic paradigms for cardiovascular disease.

## Figures and Tables

**Table 1:** Key Functions of the Vascular Endothelium.

Function	Importance	References
Physical Barrier	Forms a physical barrier between the blood and tissues, regulating the exchange of substances, including nutrients, hormones, and waste products, between the blood and cells. Endothelial cells are tightly joined, preventing pathogen infiltration.	[17,18]
Regulation of Permeability	Modifies permeability in response to stimuli, allowing immune cells to reach sites of infection or injury.	[19]
Anticoagulant and Procoagulant	Balances clot prevention and formation, playing a key role in maintaining hemostasis and preventing excessive bleeding.	[15]
Regulation of Vascular Tone	Produces vasoactive substances like nitric oxide (NO), crucial for maintaining proper blood flow and pressure.	[20]
Inflammatory Response	Produces cytokines to attract leukocytes, enabling effective immune response to infection or injury.	[21]
Interactions with Blood Cells	Interacts with various types of blood cells, such as leukocytes, platelets, and erythrocytes. These interactions are essential for processes like preventing clotting, immune responses, and gas transport.	[22]

**Table 2:** Functions of Integrins.

Function	Importance	References
Cellular Adhesion	Integrins enable endothelial cells to adhere to the ECM, maintaining the structural integrity of the endothelium and stabilizing intercellular junctions.	[26,27]
Cell Migration	Participates in endothelial migration during angiogenesis and in response to vascular injury, which is essential for tissue repair and blood vessel formation.	[28,29]
Signal Transduction	Transmits signals from the ECM to the cell interior, regulating processes such as proliferation, differentiation, survival, and response to mechanical stress.	[30,31]
Cytoskeleton Regulation	Connects with cytoskeletal proteins like actin, enabling cytoskeletal rearrangements necessary for cell migration and maintenance of cell shape.	[32,33]
Angiogenesis	Regulates adhesion and migration of endothelial cells, essential for new vessel formation during wound healing and tissue repair.	[34,35]
Vascular Permeability Regulation	Influences intercellular junction reorganization, allowing control of nutrient and leukocyte flow to tissues.	[36,37]
Mechanotransduction	Responds to mechanical forces, such as shear stress, adapting cellular functions to dynamic conditions within the vascular environment.	[38,39,40]
Interactions with Leukocytes	Facilitates leukocyte adhesion and migration through the endothelium during inflammation, playing a key role in the immune response.	[41,42,43]
Cell Survival and Apoptosis	Regulates cellular fate by activating signaling pathways that influence survival or apoptosis in response to ECM interactions.	[44,45]

**Table 3:** Integrins: Vital Receptors and Signaling Agents in Cellular Adhesion.

**Complex Structure**	Integrins are composed of 24 transmembrane heterodimers formed by 18 α and 8 β subunits, essential for cell adhesion and signaling [27]. Each heterodimer features an extracellular domain for ligand recognition, a transmembrane domain, and a cytoplasmic domain for intracellular interactions [53,54].
**Dynamic Signaling Mechanisms**	Integrins engage in both inside-out and outside-in signaling, with inside-out activation enhancing affinity for ligands, while ligand binding triggers outside-in signaling that influences downstream cellular responses. Key ligands include ECM proteins such as fibronectin, vitronectin, laminin, and collagen [55].
**Regulation and Activation Dynamics**	Activation of integrins requires intracellular proteins like talin and kindlin, which induce conformational changes from inactive to active states, facilitating ligand binding [56,57].
**Physiological and Pathological conditions**	Integrins are crucial in various physiological processes, including cell adhesion, migration, survival, and proliferation, maintaining tissue homeostasis and development. Dysregulated integrin activity contributes to numerous pathological conditions, including cancer progression, cardiovascular diseases, inflammation, and autoimmune disorders [43].

**Table 4:** Various integrin receptors and their associated ligands and cellular targets.

Receptor Type	Integrins	Distribution	Ligands Bound
Laminin Receptor [64,65]	α3β1, α6β1, α6β4, α7β1	Epithelial cells, Activated T cells	Laminins, Thrombospondin, Tenascin-C
RGD Receptors [66,67]	αIIbβ3, α5β1, αVβ1, αVβ3, αVβ5, αVβ6, αVβ8	Platelets, Fibroblasts, Epithelial cells	Fibronectin, Thrombospondin, Tenascin, Fibrinogen, Osteopontin
Collagen Receptors [68,69]	α1β1, α2β1, α10β1, α11β1	Fibroblasts, Epithelial cells, Chondrocytes	Collagens, Tenascin-C, Thrombospondin
Leukocyte-Specific Receptors [70]	αLβ2, αMβ2, αXβ2, αDβ2, αEβ7	Leukocytes, Macrophages, Dendritic cells	VCAM, MadCAM, ICAM, ADAMS

**Table 5:** Functions of Integrins in Different Vascular Segments.

Blood Vessel Type	Key Integrins Involved	Main Role of the Integrin	References
Capillaries	αvβ3, α5β1	endothelial adhesion migration, angiogenesis	[105]
Arterioles	β1	polarization, lumen formation	[106]
Arteries	α1β1, α2β1	stability, remodeling	[107,108,109]
Veins	αvβ3, α5β1	endothelial integrity, response to stress	[110,111,112]
Lymphatic Vessels	α9β1, α4β1	lymphangiogenesis, lymph flow regulation	[113,114,115]

## Data Availability

Not applicable.

## Abbreviations

AAV	Adeno-Associated Virus
CFA	Complete Freund’s Adjuvant
CHF	Chronic Heart Failure
CRISPR/Cas9	Clustered Regularly Interspaced Short Palindromic Repeats/associated protein 9
dLNs	Lymph Nodes
EAE	Experimental Autoimmune Encephalomyelitis
ECM	Extracellular Matrix
FAK	Focal Adhesion Kinase
GPCPs	G-Protein-Coupled Receptors
HFpEF	Heart Failure with Preserved Ejection Fraction
hMVEC	Human Microvascular Endothelial Cells
ICAM-1	Intercellular Adhesion Molecule 1
IL-6	Interleukin 6
LECs	Lymphatic Endothelial Cells
LFA-1	Lymphocyte Function-Associated Antigen 1
MDCK cells	Madin-Darby Canine Kidney Cells
MIDAS	Metal-Ion-Dependent Adhesion Site
MS	Multiple Sclerosis
NO	Nitric Oxide
PGA	Polyglycolic Acid
PML	Progressive Multifocal Leukoencephalopathy
RGD	Arginine-Glycine-Aspartic Acid
RTKs	Receptor Tyrosine Kinases
S1P	Sphingosine-1-Phosphate
TGF-β1	Transforming Growth Factor Beta 1
TN-C	Tenascin-C
TNF-α	Tumor Necrosis Factor Alpha
VCAM-1	Vascular Cell Adhesion Molecule 1
